# Retraction Note: Association of cord blood asprosin concentration with atherogenic lipid profile and anthropometric indices

**DOI:** 10.1186/s13098-023-01008-x

**Published:** 2023-03-09

**Authors:** Hanan Khudhair Hussein, Nassrin Malik Aubead, Hamzah H. Kzar, Yasir Salam Karim, Ali H. Amin, Moaed E. Al-Gazally, Tousief Irshad Ahmed, Mohammed Abed Jawad, Ali Thaeer Hammid, Abduladheem Turki Jalil, Yasser Fakri Mustafa, Marwan Mahmood Saleh, Hafez Heydari

**Affiliations:** 1 College of Medicine, University of Babylon, Babil, Iraq; 2 Department of Obstetrics and Gynecology, Hammurabi College of Medicine, University of Babylon, Babil, Iraq; 3 Veterinary Medicine College, Al-Qasim Green University, Al-Qasim, Iraq; 4 Al-Manara College for Medical Sciences, Maysan, Iraq; 5 Deanship of Scientifc Research, Umm Al-Qura University, Makkah, Saudi Arabia; 6 Zoology Department, Faculty of Science, Mansoura University, Mansoura, Egypt; 7 College of Medicine, University of Al-Ameed, Karbala, Iraq; 8 Department of Biochemistry, SKIMS, Srinagar, J&K India; 9 Al-Nisour University College, Baghdad, Iraq; 10 Computer Engineering Techniques, Faculty of Information Technology, Imam Ja’afar Al-Sadiq University, Baghdad, Iraq; 11 Faculty of Biology and Ecology, Yanka Kupala State University of Grodno, 230023 Grodno, Belarus; 12 College of Technical Engineering, The Islamic University, Najaf, Iraq; 13 Department of Pharmaceutical Chemistry, College of Pharmacy, University of Mosul, Mosul, 41001 Iraq; 14 Department of Biophysics, College of Applied Sciences, University of Anbar, Ramadi, Iraq; 15 Non-Communicable Diseases Research Center, Sabzevar University of Medical Sciences, Sabzevar, Iran

**Retraction: Diabetology & Metabolic Syndrome (2022) 14:74** **https://doi.org/10.1186/s13098-022-00844-7**

The Editor-in-Chief has retracted this article. After publication concerns arose regarding the authorship of this article. Contrary to the data availability statement, the authors have not provided the underlying data on the editor's request. Additionally, the authors have not been able to provide documentation that ethical approval was obtained prior to commencing this study. The ethics approval number as stated in this article appears to be identical to an ethics approval number in a previously published article by some of the same authors [1]. The Editor-in-Chief therefore no longer has confidence in the reliability of the data reported in the article. The authors did not explicitly state whether they agree to this retraction.
